# Systematic review of interventions to improve the psychological well-being of general practitioners

**DOI:** 10.1186/s12875-016-0431-1

**Published:** 2016-03-24

**Authors:** Marylou Murray, Lois Murray, Michael Donnelly

**Affiliations:** School of Medicine, Dentistry and Biomedical Sciences, Centre for Public Health, Queen’s University Belfast, Institute of Clinical Sciences, Block B, Royal Victoria Hospital, Belfast, BT 12 6BA UK; School of Public Health, Health Education North West, Regatta Place, Summers Road, Liverpool, L3 4BL UK; UKCRC Centre of Excellence for Public Health (Northern Ireland), Institute of Clinical Sciences, Royal Victoria Hospital, Belfast, BT 12 6BA UK

**Keywords:** Primary care, General practitioners, Mental health, Well-being

## Abstract

**Background:**

The health of doctors who work in primary care is threatened by workforce and workload issues. There is a need to find and appraise ways in which to protect their mental health, including how to achieve the broader, positive outcome of well-being. Our primary outcome was to evaluate systematically the research evidence regarding the effectiveness of interventions designed to improve General Practitioner (GP) well-being across two continua; psychopathology (mental ill-health focus) and ‘languishing to flourishing’ (positive mental health focus). In addition we explored the extent to which developments in well-being research may be integrated within existing approaches to design an intervention that will promote mental health and prevent mental illness among these doctors.

**Methods:**

Medline, Embase, Cinahl, PsychINFO, Cochrane Register of Trials and Web of Science were searched from inception to January 2015 for studies where General Practitioners and synonyms were the primary participants. Eligible interventions included mental ill-health prevention strategies (e.g. promotion of early help-seeking) and mental health promotion programmes (e.g. targeting the development of protective factors at individual and organizational levels). A control group was the minimum design requirement for study inclusion and primary outcomes had to be assessed by validated measures of well-being or mental ill-health. Titles and abstracts were assessed independently by two reviewers with 99 % agreement and full papers were appraised critically using validated tools.

**Results:**

Only four studies (with a total of 997 GPs) from 5392 titles met inclusion criteria. The studies reported statistically significant improvement in self-reported mental ill-health. Two interventions used cognitive-behavioural techniques, one was mindfulness-based and one fed-back GHQ scores and self-help information.

**Conclusion:**

There is an urgent need for high quality, controlled studies in GP well-being. Research on improving GP well-being is limited by focusing mainly on stressors and not giving systematic attention to the development of positive mental health.

**Electronic supplementary material:**

The online version of this article (doi:10.1186/s12875-016-0431-1) contains supplementary material, which is available to authorized users.

**How this fits in:** This review has identified a research gap in terms of mental health promotion and disease prevention interventions aimed at GPs especially those that focus on improving the positive or ‘flourishing’ concept of well-being.

## Background

Within healthcare systems the degree to which they are based on a primary care model relates positively to the delivery of efficient, cost-effective and high quality care [1]. However as the volume and complexity of clinical work increases, with concomitant rising administrative and bureaucratic burden, there are reports of rising levels of work-related stress and falling job satisfaction that raise concern about the future of primary care [2]. Top stressors identified in 2015 were increasing workloads, changes to meet the requirements of external bodies, insufficient time to do the job justice, paperwork and increasing patient demand [2]. Although most GPs report this workload as generally manageable they describe it as negatively impacting on the quality of patient care [3]. In addition to workload concerns recruitment and retention problems continue to escalate [4, 5]. The proportion of GPs intending to quit direct patient contact in the next five years continues to increase annually with 60.9 % of GPs over 50 years age reporting this intention in a recent UK survey [2].

A more pathogenic work environment appears to be developing for a population already known to be at risk of mental ill-health including burnout, depression and addiction [6–14]. Given the importance of work-related health there is a pressing need to find and appraise ways to protect and improve GP mental health.

There is a paucity of evidence on mental ill-health prevention in GPs and reviews of occupational well-being interventions have reported few studies in those working in primary care [15, 16]. A biomedical model of well-being based on a single continuum ranging from healthy worker through sickness absence to returning to work underlies most work-related health promotion [17]. Recent research developments in well-being, positive and organizational psychology [18–20] have provided an opportunity to broaden the scope of mental ill-health prevention towards the more distal concept of mental health promotion. The latter aims to create environmental conditions that empower and enable optimum health and development whilst the former aims to reduce the risk or recurrence of mental ill-health [21].

Over the past decade consensus has been emerging from leading well-being researchers as to what constitutes optimum mental health. Former advocates of either a hedonic (pleasure seeking/happiness) perspective or a eudaimonic (meaning/functioning) perspective now recognize the requirement to incorporate elements from both to capture the construct of optimum well-being or ‘flourishing’ [22–24]. This has been conceptualised as representing one end of a single continuum from mental illness [25]. Another theoretical perspective is the two continua model where languishing to flourishing represents a related but separate continuum to the presence or absence of mental illness. The resultant quadrants provide a more complete view of mental health recognising possibilities including those of positive mental health with concurrent mental illness; absence of mental illness with low positive mental health (languishing); presence of mental illness with low mental health and the optimum state of positive mental health with the absence of mental illness (flourishing). This model recognises the possibility of mental health optimisation via interventions that develop psychological resources and capacities [26–28].

There remains debate about constituent elements within flourishing. Our detailed discussion of this and the operational definition of well-being developed for the purpose of this review concluded that ‘it is a multidimensional construct that comprises the core dimensions of (i) positive affect, (ii) personal relationships and social engagement and (iii) a life view that is meaningful and optimistic’ [29].

This is the first systematic review of studies of interventions across and within the two continua of ‘mental illness to absence of mental illness’ and ‘languishing to flourishing’. This comprehensive model of well-being is best suited to our combined mental health promotion and mental illness prevention approach. The review aims to evaluate the research evidence regarding the effectiveness of interventions designed to improve GP well-being with either a mental ill health focus or a positive mental health focus or both. This comprehensive approach facilitates exploration of the extent to which research developments and reviews in positive psychology and organizational studies may be integrated within existing health to illness approaches to promote ‘flourishing’ among GPs.

## Method

The review followed the methodology specified in our PROSPERO-registered protocol and conforms to Preferred Reporting Items for Systematic Reviews and Meta-analyses (PRISMA) guidelines [30].

### Sourcing Information

A specialist subject librarian assisted in the development of a search strategy designed to identify internationally recognised terminology in peer-reviewed journals. Full details of this strategy are available in the published protocol [29].

A scoping review informed the selection of databases. Six databases were searched from inception until January 2015: Cochrane Register of Trials, MEDLINE, EMBASE, PsycINFO, CINAHL and Web of Science. Only studies published in English language peer-reviewed journals were eligible. This decision was made at the protocol stage due to concerns about potential heterogeneity in constructs across languages following operationalisation of the term ‘well-being’. (In practice the paucity of evidence identified did not merit such stringent parameters).

### Selection criteria

As we aimed to evaluate research evidence for the effectiveness of GP well-being interventions a control group was the minimum design requirement for a study to be included and only studies (including mixed-occupational group studies) in which GPs were the primary participants were eligible.

In recognition of various perspectives on well-being eligible interventions included ‘distal’ or ‘proximal’ approaches to well-being improvement. Distal-level interventions (mental health promotion) comprised, for example, strategies that promoted protective factors including the development and application of personal strengths and psychological capacities. Examples of proximal interventions (mental illness prevention) included efforts designed to promote early help-seeking behaviours, raise mental health awareness and address stigmatisation. In addition to an operational definition of well-being the protocol provided a process to resolve potential disputes in this context regarding the eligibility of interventions.

Primary outcome measures included validated tools that measured either mental illness such as the General Health Questionnaire (GHQ) [31] or positive mental health such as the Warwick Edinburgh Well-being Scale [24].

Studies designed to improve patient management by increasing GP knowledge and clinical skills enhancements were ineligible as were studies of interventions (such as rehabilitation and return-to-work programs) that were delivered at a tertiary level to GPs recovering from mental ill-health. Consensus between reviewers (MM, LM) on eligibility across all criteria was attained without arbitration from the third (MD).

In a two stage study selection process titles and abstracts were assessed independently by two reviewers (MM and LM). The study selection pilot identified 99 % agreement. The third reviewer (MD) provided additional quality control by screening 10 % of the titles during Stage 1 of the selection process. In Stage 2 full texts of studies appearing to meet inclusion criteria were independently assessed by two reviewers (MM and LM) to ascertain eligibility. Reasons for excluding studies were recorded and tabulated (see Table 1 ‘Summary Table of Excluded Studies’ and Additional file 1 ‘Full Table of Excluded Studies’).

**Table 1:** Summary of excluded studies

Reason for exclusion	Number	Studies
Population not GPs	6	Bolton 2001; Hankir 2014; O’Reilly 2007; Ospina-Kammer(from Krasner); Rahe 2002; Rowe 1999
No intervention	11	Bluestein 2011 - *Commentary* Firth-Cozens 2001 - *Proposals for interventions* Gardner 2005 - *Survey* Gutkin 2003 - *Commentary* Hansen 2013 - *Qualitative investigation of strategies* Hickner 2014 - *Commentary* Latha 2004 - *Overview of clinical environment* MacLean 2009 - *Commentary* Sim 1996 - *Commentary* Sim 1997 - *Systematic review* Taub 2006 - *Ethical guidelines*
Uncontrolled before and after study	7	Dunn 2007 (from Gardiner [34]); Fortney 2013; Gardiner 06; Krasner 2009; Manocha 2009; Margalit 2005; Winefield 1998.
Cohort study	4	Place 2013; Ro 2007; Ro 2010; Ro 2012
Qualitative evaluation	1	Schneider 2014
TOTAL	29	

### Data extraction

Data were managed using REFWORKS. The agreed data extraction form included identification features, study and participant characteristics, intervention details, outcome measures and results.

### Quality assessment

Each eligible study was appraised critically for key methodological aspects using the Cochrane Risk of Bias Tool [32] and the Quality of Assessment Tool for Quantitative Studies [33].(see ‘Risk of Bias’ and ‘Table of Quality Assessment’)

### Data synthesis

Results were organised and configured in narrative format as recommended by the experienced reviewer (MD) following detailed descriptive tabulation. The eligible studies were not considered to be of sufficiently good quality and fit to conduct a meta-analysis.

## Results

Following de-duplication 5392 studies were screened. Of the thirty-three studies that were assessed at Stage 2 twenty-nine were excluded. Main reasons for exclusion at this stage included lack of intervention and uncontrolled study design. Four studies met the methodological and design criteria for inclusion. (See Additional file 2 PRISMA Flow diagram; Table 2 Included Studies.) The total number of GP participants was 997. All of the eligible studies used outcome measures indicative of a mental ill-health focus.

**Table 2:** Included studies

Author/Year	Country	Population	Intervention	Comparator	Study design	Outcome measures/Timepoints	Numbers analysed	Results
Gardiner et al. [34]	Australia	86 GPs elected to attend a cognitive behavioural management course for which they gained Continued Professional Development (CPD) points IG *50-59 years 32.9 % * CG *50-59 years 42.9 % * Setting – metropolitan area Adelaide	15 h over 5 weeks. Cognitive behavioural management. Aims 1.To improve psychological well-being through stress reduction 2. Have a beneficial effect on coping styles 3. Improve morale through problem-focussed coping	24 GPs attending similar length CPD courses. Reported as being slightly older.	Controlled before and after study	Work-related distress (WRD -7 items Max score 49 = high distress)) Work-related morale (WRM – 7 items Max score 49 = high morale. Poor = 29) Quality of working life (QoWL – Rate statements 1-7. Max score 42. ‘Poor’ = 22) GHQ-12 (12 considered above threshold for high distress) Coping with work events (CwWE) Included to assess role of coping styles in stress outcomes Outcomes collected at T1 = Pre-intervention on first night of course T2 = Post-intervention on last night of course T3 = 12 weeks after first session	*Intervention group (IG)* T1 = 86 T2 = 77 T3 = 62 *Control group(CG*) T1 = 24 T2 = 19 *Exclusions* 0 *Withdrawals* 0 *Lost to follow-up* *IG* T2 = 9 T3 = 24 *CG* T2 = 5	WRD- Higher = more distress *IG v CG pre v post-intervention* ANOVA *F* = 2.99 *p* = 0.09 T1-T3 in IG *F* = 9.8 *p* = 0.000 WRM Rating morale as ‘poor’ *IG v CG pre v post-intervention* ANOVA *F* =2.1 *p* = 0.15 T1-T3 in IG *F* = 12.6 *p* = 0.000 QoWL Rating quality as ‘poor’ *IG v CG pre v post-intervention* ANOVA *F* =2.0 *p* = 0.16 T1-T3 in IG……*F* = 14.0 *p* = 0.000 GHQ *IG v CG pre v post-intervention* *F* = 11.9…*p* = 0.001 T1 = T3 in IG *F* = 28.2 *p* = 0.000 CwWE *IG v CG pre v post intervention* No significant difference
Gardiner et al. [34]	Australia	312 Rural GPs in reference group used to determine actual retention rate; 69 Rural GPs in intervention group who volunteered to attend a work/life balance retreat advertised by Rural Doctors’ Workforce Agency (RDWA) 205 Rural GP respondents to RDWA survey in control group Majority were male, 2/3 aged 30-50 years.	9 h Work/life balance retreat. Format Group and individual CB coaching. *Pre-retreat* – Drs’ issues, subjective stress ratings, validated stress questionnaire. *Post retreat* – letter to self at 4 weeks, e-mail follow-up and support for 5-6 weeks, Interview to assess goals at 10 weeks, validated stress questionnaire. Over 3 years 8 retreats were held.	Baseline data from RDWA survey reported in Gardiner 2005. 205/440 respondents to survey were used as the control group for the intervention. The entire population of rural GPs (312) was used to calculate actual retention rate at 42 months after the intervention	Controlled before and after study.	Rural Doctor Distress (RDD) (Customised 10 item scale graded 1-7 where 1 = not at all) Doctors’ Intention to leave rural practice (ITL) (Rated by IG on scale 1-7 where 1 = not at all) Actual retention rate of rural GPs (ARR) (Calculated by comparison of IG with de-identified data from RDWA database) Outcomes collected at T1 = Pre-intervention T2 = 3 months after intervention T3 = 42 months after intervention RDD reported T1 T2 ITL reported T1 T2 ARR reported T3	*Intervention group* (IG) *n* = 40 *Control group n* = 205 Of 69 GPs who volunteered to attend 48 completed post-intervention questionnaires T2 but only 40 were eligible due to inconsistencies in personal codes. Missing – Number analysed in intervention group at T1 not specified. CG Intention to leave at T2 = 10 IG Intention to leave at T2 = 3	Rural Doctor Distress (RDD) *IG v CG* at baseline Not significantly different *p* > 0.05 *IG* T1-T2 Significant *t*-test for all 10 items. *p* = 0.05 Doctors’ intention to leave *IG* T1-T2 Chi^2^ *p* = 0.023 % intending to leave practice *CG v IG* T1 v *IG* T2 47.5 % v 81.1 % v 40 % Actual retention rate *IG v CG* *93.9 % v 79.5 % * Chi^*2*^ *p* = 0.027
Holt & Del Mar [36]	Australia	819 GPs respondents to questionnaire 233 GPs eligible for inclusion in study as had GHQ-12 ≥ 3 Of the 819 questionnaire respondents 552 were male. No gender data on the 233 eligible for the intervention. Questionnaire sent to 1356 GPs from 8 divisions in 2 Australian states.	Aim Need for broader organizational approach to occupational stress Format Mailed intervention consisted of letter with feedback on GHQ score, interpretation of score and self-help sheet which addressed issues identified in baseline data.	Questionnaire respondents with GHQ-12 score ≥3 were divided into 2 groups. *Control group* (CG) *n* = 113 *Intervention group (IG) n* = 120	Controlled trial	GHQ-12 item Used to detect psychological distress and changes within the same population. Scores classified as 0-2 = none to mild psychological distress 3-7 = mild to moderate 8-12 = moderate to severe. Outcomes collected and reported at T1 = pre-intervention/baseline T2 = 3 months post-intervention	*Intervention group* *n* = 78 *Control group* *n* = 83 Analysed both as intention to treat and then excluding the 26 GPs who attended a concurrent educational programme. Exclusions = 14 in the Intervention group who enrolled in a concurrent educational program that used similar material. Lost to follow-up *IG* T2 = 42 *CG*T2 = 30	GHQ-12 scores Analysed by intention to treat *IG pre and post-intervention* Change = 3.39 *CG pre and post-intervention* Change = 2.25 *IG v CG* Difference of means 1.14 (0.07,2.27) *p* = 0.05 Analysed after excluding data from 26 GPs Difference of means 1.44 (0.18, 2.7) *p* = 0.03 Results for GPs attending the educational program showed that 62 % scored ≤2 on GHQ-12
Martin Asuero et al. [37]	Spain	68 Primary care professionals elected to attend a mindfulness education programme. Mean age 47 92 % women 60 % doctors 33.3 % nurses 6.7 % social workers and clinical psychologists. Intervention group1 *n* = 21 Intervention group2 *n* = 22 Authors report no significant baseline differences in intervention groups. Setting – Primary health care centres in Catalonia.	28 h over 8 weeks. Mindfulness-based group psychoeducational activities. Aims To assess the effectiveness of a training programme designed to reduce burnout and mood disturbance, to increase empathy and to develop mindfulness. Format Intervention was delivered by the same trained instructor to both intervention groups Weekly sessions included educational presentations; formal mindfulness meditation; narrative and appreciative inquiry exercises and group discussion. There was an 8 h guided silent mindfulness session. Materials/Homework Participants paid $68 for packs containing a CD recording of exercises with an explanatory booklet. Home practice was expected.	25 Primary care professionals who were offered the intervention after study completion.	Controlled clinical trial	Maslach Burnout Inventory (MBI) 22items 3 subscales. Higher scores on emotional exhaustion and depersonalization; lower scores on personal accomplishment indicate a higher degree of burnout. Possible scores 0-140 Profile of mood states (POMS) Short version- 15 items- 5 subscales: tension-anxiety, depression-dejection, anger-hostility, vigour-activity, fatigue-inertia. Higher scores indicate a worse mood state (except vigour) Scores range from 0-60 morale. Jefferson Scale of Physician Empathy (JSPE) 20 items 3 subscales. Higher scores on compassionate care, perspective taking and ‘standing in the patient’s shoes’ indicate higher degree of empathy Five facets mindfulness questionnaire (FFMQ). Observing, describing, acting with awareness, non-judging, non-reactivity rated on 5 point Likert where 1 = never/very rarely -5 = very often/always .39 items Evaluation questionnaire Translated into Spanish from University of Massachusetts Center for Mindfulness All measured at baseline T1and 8/52 post-intervention T2	*Intervention group (IG)* T1 = 43 T2 = 43 *Control group(CG*) T1 = 25 T2 = 25 *Exclusions* 0 *Withdrawals* 0 *Lost to follow-up* *IG* T2 = 0 *CG* T2 = 0	MBI *SRM IG = 0.43 CG = -0.11* *Mean between group difference* *-7 (-13.4 to -0.6 (95 % CI)) p < 0.05* *SES 0.74* POMS SRM IG = 0.62 CG = -0.1 *Mean between group difference* -0.71 (-11 to -3) *p* < 0.01 SES 1.15 JSPE SRM *IG = 0.31 CG = -0.24* *Mean between group difference* *5.2 (0.2 to 10.3)* *p < 0.05* *SES 0.71* FFMQ *SRM IG = 0.65 CG = 0.1* *Mean between group difference* *11*[3–19] *p < 0.01* *SES 0.9* SRM = standardized response mean. Calculated as mean change in score divided by the standard deviation of the change. SES = standardized effect size. Calculated as mean difference between groups divided by standard deviation of the control group. Values > 0.8 = large changes

There were two controlled before and after studies and two controlled clinical trials. The control group in Gardiner et al (2004) [34] comprised GPs who attended Continuous Professional Development (CPD) courses of similar duration but with different aims and content. Respondents to a survey carried out in 2005 by the same RDW Agency that recruited volunteer GPs for the intervention group were the comparator group in the Gardiner et al. (2013) study [35]. Some of these ‘controls’ may have volunteered subsequently to attend the intervention. The control group in the Holt and Del Mar study comprised GPs who, similar to GPs in the intervention group, had a baseline GHQ-12 ‘case’ score of ≥3 [36] Asuero et al had a wait list control group formed after stratified randomisation of primary care workers (physicians, nurses, others) recruited to attend a mindfulness education programme [37].

Two studies used the GHQ-12 [31] as the primary outcome measure. Both Gardiner et al. 2004 and Holt and del Mar 2006 report significant short-term improvement in psychological distress indicated by GHQ-12 scores despite substantial differences in duration and content of their interventions. Asuero et al. reported significant improvement in burnout, mood disturbance, empathy and mindfulness immediately post-intervention. Long-term follow-up of mental ill-health was not reported in any of the studies. No measures of positive mental health or flourishing were reported.

Interventions in both Gardiner et al. studies had a cognitive-behavioural basis. They were delivered in different formats. Cognitive-behavioural stress-management techniques were taught in 15-h over 5 weeks in Gardiner et al. (2004) while in Gardiner et al. (2013) GPs were group coached in cognitive-behavioural techniques addressing issues such a coping skills and time management during a 9-h ‘retreat’ with 5-6 weeks individual follow-up via e-mail. In contrast to that approach Holt and Del Mar used baseline GHQ results to develop a mailed brief, individually-tailored guide that interpreted GHQ scores to increase awareness about mental health risk among GP intervention ‘cases’ and provide them with self-help advice. Awareness also underpinned Asuero et al’s mindfulness based education programme. This was modelled on an earlier uncontrolled study set in primary care that reported evidence of decreased burnout and mood disturbance using mindfulness-based stress reduction principles [38]. Asuero et al delivered 28 h of group psychoeducational activities over 8 weeks with weekly sessions that included didactic presentations on awareness of thoughts, feelings, self-care and setting boundaries; formal mindfulness meditation (facilitating non-judgemental awareness);narrative and appreciative inquiry (looking for the positive in organizations by identifying current and potential processes that work well [39]) and group discussion. Consistent with original mindfulness-based stress reduction programmes this intervention also had an 8 h session of guided silent mindfulness [40].

The interventions focussed on self-awareness and amelioration of stress response consistent with a mental illness prevention approach. There were not any studies identified that appeared to be designed to promote positive mental health, flourishing. All studies reported statistically significant short-term improvement in psychological distress. Risk of bias was rated high in 4 categories for both Gardiner studies and in 2 categories for the remaining studies. Table 3 Risk of bias. Global quality rating using the Quality of Assessment tool for Quantitative Studies for all studies was weak. Tables 4 and 5.

**Table 3:** Risk of bias

Bias	Gardiner et al. [34]	Holt and Del Mar	Gardiner et al. [34]	Asuero et al. [37]
Random sequence generation (selection bias)	High – Allocation by preference of participant	Unclear –Insufficient information provided about sequence generation	High – Allocation by judgement of participant	Unclear – Insufficient information provided about sequence generation
Allocation Concealment (selection bias)	High – Explicitly unconcealed procedure	Unclear – Insufficient information provided	High – Explicitly unconcealed procedure	Unclear-Insufficient information provided
Blinding of participants and personnel (performance bias)	High – Blinding of participants and personnel was not possible	High – Blinding of participants and personnel was not possible	High – Blinding of participants and personnel was not possible	High – Blinding of participants and personnel was not possible
Blinding of outcome assessment (detection bias)	High – Self-reported outcomes	High – Self-reported outcomes	High – Self-report for Rural Doctor Distress and Intention to leave.	High Self-reported outcomes
Incomplete outcome data (attrition bias)	Unclear – Insufficient reporting of attrition to justify ‘low’ risk	Low – Clear participant flow reported	Unclear – Insufficient reporting of attrition to justify ‘low’ risk	Unclear–Baseline table indicates there were dropouts in the intervention group. No details provided
Selective reporting (reporting bias)	Low – The published report includes all expected outcomes	Low – All outcomes reported	Low – The published report includes all expected outcomes	Low-All outcomes reported.
Other bias	Unclear – Insufficient information to assess whether other important risk of bias exits	High – Concurrent educational program effecting 26 participants. 14 in intervention group did not receive the intervention as a consequence. Control group contamination possible.	Unclear – Insufficient information to assess whether another important risk of bias exits

**Table 4:** Quality assessment using EPHPP tool for quantitative studies

Components	Gardiner et al [34]	Holt and Del Mar [36]	Gardiner et al [34]	Asuero et al. [37]
Selection Bias 1. Are the individuals selected to participate likely to be representative of the target populations?	Self-referred/elected therefore using dictionary definition this scores 3 = NOT LIKELY	Participants were those respondents to a questionnaire found to score above a threshold. Questionnaire sent to all GPs in 8 Divisions of General Practice in Australia. 2 = Somewhat likely	Self-referred therefore using dictionary definition this scores 3 = NOT LIKELY	Self-referred/elected to attend. Subsequent stratified randomization reported. 2 = Somewhat likely
Selection Bias 2. What percentage of the selected individuals agreed to participate?	1 = 80-100 %. By electing to attend participants were agreeing to participate.	Baseline questionnaire response rate 819/1356 = 60 % 60 % = 2	69 Volunteered to attend but cannot tell how many actually participated 5 = Can’t tell	1 = 80-100 % All eligible volunteers agreed to participate.
SELECTION BIAS RATING	WEAK	MODERATE	WEAK	MODERATE
Study design	Controlled before and after study	Controlled clinical trial	Controlled before and after study	Controlled clinical trial
Was the study described as randomized?	No	Yes	No	Yes
Was the method of randomization described?	No	No	No	No
Was the randomization process appropriate?	Not applicable	No	Not applicable	No
STUDY DESIGN RATING	MODERATE	MODERATE	MODERATE	MODERATE
Were there important differences between groups prior to the intervention?	1 = Yes Control group more likely to be in solo practice, older and had more years in practice	3 = Can’t tell Authors report mean comparison of baseline GHQ scores showed no significant difference prior to the intervention (p = 0.09). No other information provided on pre-intervention confounders	3 = Can’t tell. Control group for psychological well-being outcome were respondents to a survey. Control group for actual retention were entire population of rural GPs .	3 = Can’t tell Authors report that intervention group was larger due to high interest in the intervention.
What percentage of relevant confounders were controlled?	Can’t tell = 4 Controlling for confounders not explicit.	Can’t tell = 4	Can’t tell = 4	Can’t tell = 4
CONFOUNDERS RATING	WEAK	WEAK	WEAK	WEAK
Were the outcome assessors aware of the intervention status of participants?	Yes = 1	Yes = 1	Yes = 1	Yes = 1
Were the participants aware of the research question?	Yes = 1	Yes = 1	Yes = 1	Yes = 1
BLINDING RATING	WEAK	WEAK	WEAK	WEAK
Were data collection tools shown to be valid?	Yes = 1	Yes = 1	Yes = 1	Yes = 1
Were data collections tools shown to be reliable?	Yes = 1	Yes = 1	Yes = 1	Yes = 1
DATA COLLECTION RATING	STRONG	STRONG	STRONG	STRONG
Were withdrawals and drop-outs reported in terms of numbers/reasons?	Yes = 1	Yes = 1	No = 2 69 volunteers, 48 questionnaires completed post-intervention. No information on those 21 given.	No = 2 Drop-outs from intervention group mentioned in baseline table. No details provided however results in scales approximate in remainder of tables.
Percentage of participants completing the study	84 % = 1 89 % IG 79 % CG	161/233 = 69 % = 2	57 % = 3 63 % IG 51 % CG	100 % = 1
WITHDRAWALS AND DROP OUTS RATING	STRONG	MODERATE	WEAK	STRONG
What percentage of participants received the allocated intervention?	Follow-up data for 77. Cannot tell if all 86 received the intervention.	106/120 = 88 % Score = 1	48/68 = 60 % Score = 2	100 % Score = 1
Was the consistency of the intervention measured?	Not explicitly Cannot tell = 3	Not explicitly Cannot tell = 3	Not explicitly Cannot tell = 3	Described as ‘*essentially the same’* and delivered by the same qualified instructor. No explicit report of measurement of consistency. Cannot tell = 3
Is it likely that subjects received an unintended intervention that may influence results?	No = 5	Yes = 4 Concurrent educational programme which 26 of the study participants attended. Analyses were made with and without them.	No = 5	No = 5
Unit of allocation	Individual	Individual	Individual	Individual
Unit of analysis	Individual	Individual	Individual	Individual
Are the statistical methods appropriate for the study design?	Yes = 1	Yes = 1	Yes = 1	Yes = 1
Is the analysis performed by intervention allocation status (ITT) rather than actual intervention received?	No = 2	Yes = 1	No = 2	No = 1

**Table 5:** Summary of Global rating for Quality using EPHPP Quality Assessment tool for Quantitative Studies

Component	Gardiner et al [34]	Holt and Del Mar [36]	Gardiner et al [34]	Asuero et al. [37]
Selection Bias	Weak	Moderate	Weak	Moderate
Study Design	Moderate	Moderate	Moderate	Moderate
Confounders	Weak	Weak	Weak	Weak
Blinding	Weak	Weak	Weak	Weak
Data Collection Methods	Strong	Strong	Strong	Strong
Withdrawals and Dropouts	Strong	Moderate	Weak	Strong
GLOBAL RATING	WEAK	WEAK	WEAK	WEAK

Criteria for global rating; 1. Strong = no weak ratings 2. Moderate = one weak rating, 3. Weak = two or more weak ratings

## Discussion

### Summary

Our review aimed to evaluate systematically the research evidence regarding the effectiveness of interventions designed to improve GP well-being with either a mental ill health focus or a positive mental health focus or both and to explore the nature and extent to which research developments in positive mental health may be integrated within existing ‘illness to health’ approaches to promote ‘flourishing’ among GPs. It identified a paucity of evidence across the mental ill-health continuum and no studies specifically designed to effect change within the positive mental health continuum. The focus was mainly on mental illness prevention rather than mental health promotion. All studies were assessed as high risk of bias using the Cochrane Risk of Bias tool. The Quality Assessment Tool for Quantitative Studies (recommended for use in non-randomised intervention studies [41]) deemed them to be ‘weak’ in quality.

The findings reported in the four included studies suggest that cognitive-behavioural-based and mindfulness–based programmes delivered in a group format may reduce GP distress at least in the short-term. Increasing awareness generally and with specific regard to thoughts, beliefs, self-care, personal health and setting boundaries appeared to improve GP mental health. Potential mechanisms include the support afforded by professional peer-groups; cognitive-behavioural techniques that address emotional distress by modifying ‘maladaptive’ thoughts and thought patterns [42] and strengthening personal resources for optimising health through mindfulness practices [40].

### Well-being interventions in healthcare professionals

The development of potentially effective well-being interventions for GPs currently requires exploration of evidence within other occupational groups. A recent Cochrane review of occupational stress interventions for healthcare workers (defined by them as any worker employed in a healthcare setting such a nurses and doctors including medical and nursing students) found that cognitive-behavioural training (approximately one third of the 58 studies) had relatively poor impact reducing stress by only 13 % compared to no intervention over periods from one month to two years [15]. Only 5 % of studies included medical doctors and there were not any GPs. Delivery to a group over circa 6 weeks was the usual format. Coping skill enhancement was a common ingredient. Other interventions included guided relaxation in various forms (*n* = 21) and organizational changes (*n* = 20). The review found low-moderate quality evidence for both physical relaxation (e.g. massage) and mental relaxation (e.g. mindfulness) - stress levels were reduced by 23 % compared to controls. Although intervention heterogeneity precluded precise identification of potentially active ingredients, these results suggest that approaches that address cognitions and relaxation techniques merit further study.

Further evidence of the potential benefit of a mindfulness-based approach was identified in an additional study which met all our inclusion criteria except the English language restriction as it was reported in Spanish. It was included in a meta-analysis which found that cognitive, behavioural and mindfulness-based interventions significantly reduce stress in doctors [43]. This RCT-evaluated, 10-week mindfulness-based intervention reported significant reduction in stress and anxiety among Spanish GPs that persisted at six-month follow-up [44]. Four of the 12 studies in this meta-analysis involved medical students (who also reported a significant reduction in stress). Most interventions were mindfulness-based and directed at hospital-based doctors.

Elucidating ‘what works’ to improve doctor well-being is difficult due to the paucity of studies. Comparisons between, for example, medical students and experienced clinicians, physicians and nurses, and primary- and secondary-care doctors provide only limited insights due to pre-existing significant differences. Arbitrary categorisation of intervention type, relatively small sample sizes and simple study designs makes it difficult to achieve clarity and certainty regarding essential active ingredients and mechanisms of effect across various intervention approaches. Whilst there is scope within e.g. mindfulness approaches for mental health promotion the emphasis in healthcare professional well-being interventions appears to be on mental illness prevention (psychopathology continuum). There is negligible evidence within this population for interventions designed to empower and enable optimum mental health through the development of personal resources thereby promoting flourishing.

### Organizational approaches to well-being

The creation of empowering work environments through organisational-change interventions has received even less research attention than person-directed interventions (focussing on individuals). Organizational approaches to mental health promotion include enhancing the flexibility of working hours [45, 46], implementation of anti-bullying policies [47, 48] and leadership training [49, 50]. Despite sound theoretical underpinnings, empirical evidence for organizational interventions remains limited.

The aforementioned Cochrane review identified 21 study arms examining the effect of organizational change in preventing occupational stress in healthcare workers. These included changing working conditions, provision of support and mentoring, communication skills training and improving work schedules. Shorter or interrupted work schedules were found to decrease stress levels however no clear benefit of other interventions was identified. They concluded that organizational interventions should be more focussed on addressing specific stressors [51].

Empirical evidence for organizational approaches is limited and often includes individual-based approaches. In a review of ‘burnout prevention’ interventions for various occupational groups, only 2/25 interventions were organizational in nature and focus [16]. Cognitive-behavioural therapy (CBT) was the single most common intervention (6/25). Only four of the seventeen person-directed interventions produced sustained benefit up-to-one-year compared to five of the six combined (person-directed and organizational) interventions suggesting that workplace mental health programmes should include an integrated approach.

### Resilience

Resilience enhancement is common to both organizational and person-directed interventions. Furthermore it can be integral to both mental health promotion and mental illness prevention programmes. Definition of resilience and specification of what constitutes resilience training remain topics of considerable debate [52–55]. A recent review of resilience in healthcare workers defined it as ‘the ability to maintain personal and professional well-being in the face of ongoing work stress and adversity’ [56]. (That review did not identify any interventions designed to increase resilience in doctors.) Organisational interventions tend to develop a ‘psychosocial safety climate’ that comprises clearly communicated managerial participation and commitment to, and prioritization of, employee psychological health [57]; enhancement of (procedural and relational) organizational justice [58] and team-based interventions to promote mental resources and resilience [59]. Research is sparse regarding an organizational, integrated systems approach to addressing doctors’ potential stressors [60]. Although person-directed resilience training has been recommended to proactively prepare doctors for ‘inevitable’ stressors [61], a distal-focused approach may be more appropriate [62].

### Application of positive psychology

Research on improving GP well-being is limited by focusing mainly on stressors and not giving systematic attention to aspects of well-being such as positive affect, engagement and optimism. The application of interventions to promote flourishing - so-called positive psychology interventions (PPIs) - in occupational groups is under-researched.

The (only) systematic review of PPIs in organizations found that 13/15 had positive effects across 29 measures of well-being including positive emotions, optimism, resilience and life satisfaction (though most investigated an individual-level outcome) [63] The only primary care-based study in this review used appreciative inquiry - a largely qualitative method of organisational change management and quality improvement [64]. They found some evidence of well-being improvement in the GPs as they developed a shared sense of purpose and increased engagement with the organisational change intervention through the implementation of change objectives. Time shortage among GPs was cited as a possible explanation for the limited success of the intervention. Appreciative inquiry may prove to be effective in the development of future GP well-being interventions.

Shifting from the deficit approach that underpins stress response amelioration towards a more proactive mental health promotion approach that empowers and enhances work and personal resources may prove to be more effective in and appropriate to our population of interest [65, 66].

### Limitations and strengths

This review is limited by an English language restriction applied at protocol stage to address potential heterogeneity in well-being terminology and constructs. However, it was conducted using robust methodology and identified a substantial research gap. It is the first review to evaluate an extensive body of research pertinent to the optimisation of well-being in GPs.

## Conclusion

The evidence base in this area is limited. There is a clear need for pragmatic randomised controlled trials using validated assessments of the positive construct of well-being to identify strategies that will help safe-guard the mental health of doctors working in primary care.

## Additional files

Additional file 1: Full Table of Excluded Studies. (DOCX 18 kb) Additional file 2: PRISMA Flow Diagram. (DOCX 19 kb)

## Abbreviations

GP: general practitioner
PPI: positive psychology interventions
CPD: continuous professional development
