# Supplementary material for: Systematic review of interventions to improve the psychological well-being of general practitioners
Source: BMC Fam Pract. 2016 Mar 24;17:36. doi: 10.1186/s12875-016-0431-1 (PMC4806499; doi:10.1186/s12875-016-0431-1)
Supplement: Additional file 1: — Full Table of Excluded Studies. (DOCX 18 kb) [file 12875_2016_431_MOESM1_ESM.docx]

**Additional file 1 Full Table of Excluded Studies**

| **Refworks No./Author/Year** | **Reason Ineligible** | **Abstract** | **Full paper** | **Data Extraction** |
| --- | --- | --- | --- | --- |
| 47  Adams E 2010 | Survey | x |  |  |
| 55  Aelfers 2013 | Population not GPs  Protocol | x |  |  |
| 103  Allamani 2010 | Commentary. No intervention | x |  |  |
| 142  Anderson P 2004 | Educational/clinical skills intervention | x |  |  |
| 195  Arnetz et al 2002 | Population not GPs  Hospital doctors | x |  |  |
| 211  Ashworth  1999 | Survey | x |  |  |
| 254  Bailie R 1998 | Survey | x |  |  |
| 270  Baldwin 1997 | Longitudinal cohort | x |  |  |
| 285  Barfod S 2008 | No intervention  A GP’s opinion | x |  |  |
| 312  Bashir K 2000 | Clinical skills intervention | x |  |  |
| 465  Blacklock 2012 | Population not GPs | x |  |  |
| 467  Blair S 1998 | Population not GPs | x |  |  |
| 476  Blashki 2003 | Not intervention  GP educational need | x |  |  |
| 478  Blashki 2008 | Educational intervention | x |  |  |
| 487  Blomstrand 2005 | Population not GPs | x |  |  |
| 489  Blount 2009 | Not intervention | x |  |  |
| 490  Bluestein 2011 | Not intervention |  | x |  |
| 511  Bolton 2001 | Letter about recruitment by GPs.  Rec’d 19.3.2015 |  | x |  |
| 586  Braumann 01 | Survey | x |  |  |
| 992  Cooper 89 | Survey  No intervention | x |  |  |
| 1259  Dory et al 09 | No intervention | x |  |  |
| Dunn 07  Cited by Gardiner 2013 |  |  | x | No control |
| 1315  Earle et al 05 | Assessment depression/anxiety  No WBI | x |  |  |
| 1493  Firth-Cozens | No study/intervention |  | x |  |
| 1538  Fortney et al 2013 |  |  | x | No control |
| 1544  Fox et al 09 | No intervention | x |  |  |
| 1646  Gardiner et al 05 | No intervention |  | x |  |
| 1647  Gardiner et al 06 |  |  | x | No control |
| 1648  Gardner 05 | No well-being intervention.  Survey | x |  |  |
| 1686  George et al 2014 | No intervention | x |  |  |
| 1697  Gerrity 01 | No intervention | x |  |  |
| 1888  Gutkin 2003 | No intervention  Received from library 18.3.2015 |  | X |  |
| 1942  Hankir2014 | Not GPs |  | x |  |
| 1951  Hansen | No intervention |  | x |  |
| 2060  Heim 1993 | No intervention. | x |  |  |
| 2100  Hickner | No intervention  Commentary  Received in post 14.3.2015 |  | x |  |
| 2155  Holt et al 2005 | Observational study | x |  |  |
| 2644  Krasner et al 2009 |  |  | x | No control. |
| 2741  Latha | No intervention  Received from library 17.3.2015 |  | x |  |
| 2795  Lemaire et al 2010 | Not GPs | x |  |  |
| 2830  Levinson et al 1993 | Educational intervention | x |  |  |
| 2950  Luce et al 2002 | Survey | x |  |  |
| 2986  Mac Lean 2009 | No intervention  Commentary Received in post 13.3.2015 |  | x |  |
| 3018  Malcolm 07 | Not GPs | x |  |  |
| 3044 Manocha et al 2009 |  |  | x | No control |
| 3052  Margalit et al 2005 |  |  | x | No control |
| 3108  Matsumoto 04 | Survey. No intervention | x |  |  |
| 3206 Meland et al 1996 | Not GPs | x |  |  |
| Ospina Kammer | Not qualified GPs. Residents. Ref from Krasner |  | x |  |
| 3811  Pit&Hansen 2014 | Cross sectional study | x |  |  |
| 3816  Place & Talen 2013 | Cohort study without any results  Rec’d in post 14.3.2015 |  | x |  |
| 3884  Putnik 2011 | Survey | x |  |  |
| 3912  Rahe 2002 | Not GPs  Rec’d in post 14.3.2015 |  | x |  |
| 3935  Rashid 2009 | Not GPs | x |  |  |
| 3937  Ratanawongsa  2008 | Study to assess clinical skill/patient care | x |  |  |
| 4040  Ro 2007 | Cohort study |  | x |  |
| 4042  Ro 2012 | Cohort study |  | x |  |
| 4043  Ro 2010 |  |  |  | Cohort study  No control |
| Rowe 99  Cited Ro 2010 | Requested from library.  Not GPs |  | x |  |
| 4141  Rosvold 2002 | Survey | x |  |  |
| 4332  Schneider 2014 | Qualitative evaluation of physician coaching. |  | x | No control |
| 4495  Sims 1997 | Not GPs |  | x |  |
| 4499  Sims 1996 | Commentary, not intervention |  | x |  |
| 4669  Strachan 2007 | Not GPs | x |  |  |
| 4685  Su 2009 | Not GPs | x |  |  |
| 4767  Taub 2006 | No intervention |  | x |  |
| 5210  Wilkinson 02 | Not GPs | x |  |  |
| 5229  Williamson 07 | Recruitment case study | x |  |  |
| 5247  Winefield 98 | WBI for female GPs |  | x | No control |
| 5250  Winefield 07 | Not GPs | x |  |  |
| 5300  Wurst 11 | Survey. Not GPs | x |  |  |
| 5329  Yelin 1996 | GP educational intervention for patient care | x |  |  |
| Totals |  | Abstract only 46 | Full Paper  32 | Excluded at data extraction 9 |
