# Supplementary material for: Systematic review of interventions to improve the psychological well-being of general practitioners
Source: BMC Fam Pract. 2016 Mar 24;17:36. doi: 10.1186/s12875-016-0431-1 (PMC4806499; doi:10.1186/s12875-016-0431-1)
Supplement: Additional file 2: — PRISMA Flow Diagram. (DOCX 19 kb) [file 12875_2016_431_MOESM2_ESM.docx]

**Additional File 2.**

**PRISMA Flow Diagram**

Full-text articles assessed for eligibility

n=33

Studies included in qualitative synthesis

n=4

Full-text articles excluded, with reasons

Total n=29
Population not GPs n =6

No Intervention n= 11

Uncontrolled before and after Study n= 7

Cohort Study n= 4

Qualitative evaluation n= 1

Medline 972

PschINFO 411

Embase 2423

Cinahl 551

Web of Science 2450

Records excluded
n=5359

Records screened
n=5392

Records after duplicates removed
n=5392

Additional records identified through other sources
n=4(references 3;expert 1)
